# A Different Pattern of Production and Scavenging of Reactive Oxygen Species in Halophytic *Eutrema salsugineum (Thellungiella salsuginea)* Plants in Comparison to *Arabidopsis thaliana* and Its Relation to Salt Stress Signaling

**DOI:** 10.3389/fpls.2016.01179

**Published:** 2016-08-04

**Authors:** Maria Pilarska, Monika Wiciarz, Ivan Jajić, Małgorzata Kozieradzka-Kiszkurno, Petre Dobrev, Radomíra Vanková, Ewa Niewiadomska

**Affiliations:** ^1^The Franciszek Górski Institute of Plant Physiology – Polish Academy of SciencesKraków, Poland; ^2^Department of Plant Physiology and Biochemistry, Faculty of Biochemistry Biophysics and Biotechnology, Jagiellonian UniversityKraków, Poland; ^3^Department of Plant Cytology and Embryology, University of GdańskGdańsk, Poland; ^4^Institute of Experimental Botany AS CRPrague, Czech Republic

**Keywords:** chloroplast, glucosinolates, halophyte, hydrogen peroxide, salinity, stress hormones

## Abstract

Isolated thylakoids from halophytic *Eutrema salsugineum* (*Thellungiella salsuginea*) produces more H_2_O_2_ in comparison to glycophytic *Arabidopsis thaliana*. The first objective of this study was to verify whether this feature is relevant also to the intact chloroplasts and leaves. Enhanced H_2_O_2_ levels in chloroplasts and leaves of *E. salsugineum* were positively verified with several methods (electron microscopy, staining with Amplex Red and with diaminobenzidine). This effect was associated with a decreased ratio of $O_{2}^{•–}$/H_2_O_2_ in *E. salsugineum* in comparison to *A. thaliana* as detected by electron paramagnetic resonance method. As a next step, we tested how this specific ROS signature of halophytic species affects the antioxidant status and down-stream components of ROS signaling. Comparison of enzymatic antioxidants revealed a decreased activity of ascorbate peroxidase (APX), enhanced activity of glutathione peroxidase, and the presence of thylakoid-bound forms of iron superoxide dismutase (FeSOD) and APX in *E. salsugineum*. These cues were, however, independent from application of salt stress. The typical H_2_O_2_-dependent cellular responses, namely the levels of glucosinolates and stress-related hormones were determined. The total glucosinolate content in *E. salsugineum* water-treated leaves was higher than in *A. thaliana* and increased after salinity treatment. Treatment with salinity up-regulated all of tested stress hormones, their precursors and catabolites [abscisic acid (ABA), dihydrophaseic acid, phaseic acid, 1-aminocyclopropane-1-carboxylic acid, salicylic acid, jasmonic acid, *cis*-(+)-12-oxo-phytodienoic acid and jasmonoyl-L-isoleucine] in *A. thaliana*, whereas in *E. salsugineum* only a stimulation in ethylene synthesis and ABA catabolism was noted. Obtained results suggest that constitutively enhanced H_2_O_2_ generation in chloroplasts of *E. salsugineum* might be a crucial component of stress-prepardeness of this halophytic species. It shapes a very efficient antioxidant protection (in which glucosinolates might play a specific role) and a fine tuning of hormonal signaling to suppress the cell death program directed by jasmonate pathway.

## Introduction

Reactive oxygen species (ROS) are intriguing molecules, which are toxic to the biological structures but also play a signaling role in controlling plant growth, development and stress responses (for a recent review, see Suzuki et al., 2012; Baxter et al., 2014). They accompany the basal metabolic fluxes of aerobic organisms during the whole ontogeny. Considering the precise and multilevel control of metabolic changes it might not be surprising that ROS coming from the various cellular and extracellular sites seem to have their specific signaling targets (op den Camp et al., 2003; Avsian-Kretchmer et al., 2004; Gadjev et al., 2006; Geisler et al., 2006; Laloi et al., 2007). In plants several ROS, such as H_2_O_2_, $O_{2}^{•–}$ and ^1^O_2_, are formed in chloroplasts aside the photosynthetic electron transport. They, in turn, affect the nuclear gene expression to adjust the photosynthesis to changing environment. The most stable form, which is assumed to leave this organelle and evoke the effects on the nuclear genes, is H_2_O_2_ (Mubarakshina et al., 2010; Borisova et al., 2012).

In an attempt to recognize the signaling effects of H_2_O_2_ originating from chloroplasts, *Arabidopsis* mutant overexpressing glycolate oxidase in chloroplasts (GO5 plants) has been developed (Fahnenstich et al., 2008). In the photorespiratory conditions GO5 mutants produce H_2_O_2_ in chloroplasts instead of in peroxisomes. Transcript profiling of GO5 plants (Balazadeh et al., 2012) identified the H_2_O_2_ regulated genes and transcription factors, whereas further work of Sewelam et al. (2014) underpinned the top 20 genes specifically up-regulated by H_2_O_2_ produced in chloroplasts. These studies proved that H_2_O_2_ can trigger different responses depending on the subcellular site of its production.

Recently, we demonstrated that thylakoids isolated from a highly stress resistant species *Eutrema salsugineum (Thellungiella salsuginea)* are capable of the enhanced production of H_2_O_2_, in comparison to *Arabidopsis thaliana*, already in the absence of stress (Wiciarz et al., 2015). This creates the opportunity to unravel the signaling action of chloroplast H_2_O_2_ generation in the natural system. *E*. *salsugineum* tolerates extreme salinity, cold, drought, ozone and over the last years this species became a plant model of stress resistance well-comparable with the *Arabidopsis* genome (Inan et al., 2004; Li et al., 2006; Amtmann, 2009; Hou and Bartels, 2015). So far, several studies focused on the discovery of transcriptomic footprints of high stress resistance in *E. salsugineum*. These studies showed that several stress-associated genes in *E. salsugineum* have a constitutively higher expression in comparison with *A. thaliana* already in the absence of stress (Inan et al., 2004; Taji et al., 2004; Gong et al., 2005). The group of up-regulated genes includes, for example, those involved in abscisic acid (ABA) biosynthesis and signaling (Taji et al., 2004; Gong et al., 2005). In contrast, after stress treatment, only a slight change in gene expression was detected in *E. salsugineum* in comparison with a great activation of transcription in *A. thaliana* plants (Taji et al., 2004; Li et al., 2006; Wong et al., 2006). Also, a comparative proteomics of *A. thaliana* and *E. salsugineum* salt responses revealed more changes in protein abundance in *Arabidopsis* than in *Eutrema* (Pang et al., 2010). Combined, these results indicate so called ‘stress preparedness’ of *E. salsugineum*, which supports its halophytic nature. Comparison of metabolite profiles of these two species following salt stress revealed a significant differences (Arbona et al., 2010). This suggests, that an adjustment of metabolism and activation of the already present enzymatic machinery serves as a faster and more efficient strategy to cope with stress than synthesis of new proteins.

A goal of this study was to verify whether enhanced generation of H_2_O_2_ in thylakoids of halophytic *Eutrema* in comparison to glycophytic *A. thaliana* is significant also *in vivo*. As a next objective, it was tested how this situation influences oxidative damage, antioxidant system and hormonal signaling in the control conditions and after a salinity stress.

## Materials and Methods

### Plant Material and Growth Conditions

*Arabidopsis thaliana* (Col-0) and *E. salsugineum* (*T. salsuginea*, salt cress) ecotype Shandong were grown from seeds in the soil culture under irrigation with tap water. Seeds were obtained from the Nottingham *Arabidopsis* Stock Centre, UK. Plants were cultivated in the phytotron chamber at temperatures of 18°/16°C day/night, photoperiod 10/14 h, irradiance of ab. 220 μmol m^-2^ s^-1^ and RH ∼50%. Both species were adapted to these conditions for at least three generations. Because of delay in growth of *E. salsugineum* in comparison to *A. thaliana*, as reported earlier (Inan et al., 2004; Stepien and Johnson, 2009), to compare rosettes at the same developmental stage, 4-weeks-old *A. thaliana* and 5-weeks-old *E. salsugineum* plants were taken for experiments. To evoke a mild salinity-stress plants were irrigated with 0.15 and 0.3 M NaCl solutions for *A. thaliana* and *E. salsugineum*, respectively, while watered plants served as controls. After 7 days of NaCl treatment complete rosettes were collected, frozen in liquid nitrogen and stored at -80°C until further use (unless stated otherwise).

### Thylakoid Membrane Preparation

Thylakoids were isolated as described earlier (Wiciarz et al., 2015). Shortly, leaves were homogenized in medium containing 50 mM HEPES-KOH (pH 7.6), 330 mM sorbitol (control plants) and 495 mM sorbitol (NaCl-treated plants) respectively, 1 mM MgCl_2_, 2 mM Na_2_EDTA, 5 mM sodium ascorbate and 0.01% (w/v) fatty acid-free bovine serum albumine. After centrifugation for 4 min at 4000 × *g* the pellet was resuspended in 50 mM HEPES-KOH (pH 7.6), 5 mM sorbitol, 5 mM MgCl_2_ and centrifuged again. Then the pellet was washed and resuspended in 50 mM HEPES-KOH (pH 7.6), 330 mM sorbitol, 10 mM MgCl_2_, 20 mM NaCl, 2.5 mM Na_2_EDTA, 10 mM NaHCO_3_. The chlorophyll concentration was estimated spectrophotometrically according to Lichtenthaler and Buschmann (2001).

### Electron Paramagnetic Resonance (EPR) Measurements

Production of $O_{2}^{•–}$ and H_2_O_2_ by thylakoids from *A. thaliana* and *E. salsugineum* water-treated plants was detected by electron paramagnetic resonance (EPR) spin-trapping spectroscopy using DMPO (5,5-dimethyl-pyrroline *N*-oxide; Sigma-Aldrich, USA) and POBN [α-(4-pyridyl-1-oxide)-*N*-tertbutylnitrone; Sigma-Aldrich] as the spin trap, respectively, as described earlier (Jajić et al., 2015). Shortly, for $O_{2}^{•–}$ detection, isolated thylakoids (chlorophyll concentration 200 μg mL^-1^) were mixed with DMPO to a final concentration 50 mM, transferred to a flat cell and illuminated for 5 min at 500 μmol quanta m^-2^ s^-1^ within the EPR spectrometer MiniScope MS300 (Magnettech GmbH, Germany). For H_2_O_2_ detection, the H_2_O_2_-derived hydroxyl radical after the initiation of the Fenton reaction was measured. Isolated thylakoids (concentration of chlorophyll 150 μg mL^-1^) in a reaction medium pH 7.6 containing 0.4 M sucrose, 20 mM NaCl, 5 mM MgCl_2_, 10 mM Hepes-KOH were illuminated with white light source LS2 (Hansatech, UK) for 3 min at 500 μmol quanta m^-2^ s^-1^. Next, 50 mM POBN, 50 μM FeEDTA and 4% ethanol were added and incubated for 3 min, transferred to glass capillaries and measured using an EPR spectrometer. To check the influence of enzymatic scavengers on H_2_O_2_ production, measurements were performed in presence and absence of 5 mM sodium azide (inhibitor of heme containing enzymes).

### H_2_O_2_ Detection

Ultrastructural localization of H_2_O_2_ was visualized in transmission electron microscopy (TEM) via electron-dense precipitates of cerium perhydroxides [Ce(OH)_2_OOH and Ce(OH)_3_OOH] formed after the reaction of cerium chloride (CeCl_3_) with endogenous H_2_O_2_ (Bestwick et al., 1997). After 1 h of light leaves of *A. thaliana* and *E. salsugineum* from water-treated plants were cut into small pieces (∼5 mm). Leaf fragments were immediately infiltrated and then incubated for 1 h with a 0.5 M morpholinepropanesulfonic acid (MOPS) buffer (pH 7.0), containing 5 mM CeCl_3_ (Libik-Konieczny et al., 2015). The controls were tissue samples incubated in MOPS buffer. Then, tissues were quickly washed in the 0.5 M MOPS and fixed in 2.5% (w/v) formaldehyde (prepared from paraformaldehyde) and a 2.5% (v/v) glutaraldehyde in 50 mM cacodylate buffer (pH 7.0) for 4 h at room temperature. The procedure for preparing the samples for TEM was as described earlier (Kozieradzka-Kiszkurno and Płachno, 2012). The material was dehydrated in a series of graded acetone and embedded in Spurr Low-Viscosity Embedding Kit (Polysciences, Germany). Ultrathin (60–90 nm) sections were cut with a diamond knife on a Leica EM UC7 ultramicrotome. The sections were stained with uranyl acetate and lead citrate and then viewed using a Philips CM 100 TEM at 75 kV.

Histochemical localization of H_2_O_2_ production in leaves was determined using the DAB (3,3-diaminobenzidine) staining technique according to Libik-Konieczny et al. (2015). Leaves of *E. salsugineum* and *A. thaliana* water-treated plants were infiltrated with a solution of 1 mg mL^-1^ DAB (Sigma-Aldrich) prepared in water. Incubation was carried for 4 h in the dark at room temperature.

### SDS-PAGE and Immunoblot Analysis

Leaf soluble proteins were extracted with 0.1 M phosphate buffer pH 7.5 containing 1 mM dithiothreitol, 2% (w/v) polyvinylpolypyrrolidone and protease inhibitor cocktail (Sigma-Aldrich). Homogenates were centrifuged 10 min at 10000 × *g*. Protein concentration in supernatant was estimated using Roti^®^-Nanoquant Protein quantitation assay (Carl Roth, Germany). SDS-PAGE as well as immunoblotting were performed as described earlier (Niewiadomska et al., 2009). Thylakoid membranes (TMs) and soluble proteins were dissolved in denaturating buffer and heated 20 min at 99°C (soluble proteins) or 5 min at 75°C (TMs). After electrophoresis, the separated proteins were blotted onto nitrocellulose membranes and probed with polyclonal antibodies raised against: ascorbate peroxidase (APX; anti-APX); glutathione peroxidase (GPX; anti-GPX); iron superoxide dismutase (FeSOD; anti-FeSOD); gamma glutamylcysteine synthase, γ -ECS (anti-γ -ECS); peroxiredoxins Q, PrxQ (anti-PrxQ). All antibodies were purchased from Agrisera (Sweden).

### Determination of Lipid Peroxidation

Lipid peroxidation in leaves and isolated thylakoids of *A. thaliana* and *E. salsugineum* water-treated plants was assessed by measuring the malondialdehyde (MDA) content using high-performance liquid chromatography (HPLC) as described by Rastogi et al. (2014). Shortly, leaf samples were ground in a mortar with chilled 80% (v/v) ethanol. The extract was then centrifuged (10000 × *g* for 2 min) and the supernatant was further used. Samples were mixed with equal amount of reaction mixture containing 20% (w/v) trichloroacetic acid, 0.01% (w/v) butylated hydroxytoluene and 0.65% (v/v) thiobarbituric acid (TBA). After heating at 95°C for 20 min and centrifugation the MDA-(TBA)_2_ adduct was separated and quantified by the HPLC. The elution buffer was 50 mM KH_2_PO_4_ (pH 7.0)/methanol (65:35, v/v). The retention time was 5 min and flow rate of 0.5 mL min^-1^ with detection at 530 nm. Tetraethoxy-propane (Sigma-Aldrich) was used as a standard.

### Quantification of H_2_O_2_ Concentration

An Amplex^®^ Red Hydrogen Peroxide/Peroxidase Assay Kit (Life Technologies/Thermo Fisher Scientific, USA) was used to measure H_2_O_2_ content in *A. thaliana* and *E. salsugineum* leaves after water irrigation. Leaves (0.1 g) frozen in liquid nitrogen were ground with 0.5 M sodium phosphate buffer, pH 7.4, and centrifuged 10 min at 10,000 × *g*. Proteins were removed from the extracts using Amicon Ultra Centrifugal Filters (Merck Millipore, USA) and the filtrate (25 μL) was incubated for 30 min in dark conditions with 50 mM Amplex Red reagent and 0.2 units mL^-1^ horseradish peroxidase. Excitation was measured at 530 nm and fluorescence detection at 590 nm with a microplate reader Synergy 2 (BioTek, USA). The hydrogen peroxide concentration was estimated by comparison with standard curve (0–10 μM) of H_2_O_2_. The experiments were repeated three times independently, each time in triplicate.

### Determination of Antioxidant Enzyme Activities

Soluble leaf proteins were extracted with 0.1 M phosphate buffer pH 7.5 [catalase (CAT); SOD], 50 mM phosphate buffer pH 7.0 (GPX) or 0.1 M phosphate buffer pH 7.8 with 1 mM ascorbic acid (APX), in each case containing protease inhibitor cocktail (Sigma-Aldrich). After centrifugation 10 min at 10000 × *g* at 4°C soluble proteins were desalted using Amicon Ultra Centrifugal Filters (Merck Millipore). Protein concentration was estimated using Roti^®^ -Nanoquant Protein quantitation assay (Carl Roth).

Catalase activity was measured according to the method described by Aebi (1984). The decomposition of 10 mM H_2_O_2_ in phosphate buffer (50 mM phosphate buffer pH 7.0) was monitored for 2 min at 240 nm. Calculations used an absorbance coefficient of 43 M^-1^ cm^-1^.

Ascorbate peroxidase activity was determined according to Nakano and Asada (1981). The decrease in absorbance at 290 nm due to ascorbate oxidation was recorded. The assay mixture contained 20 μg proteins, 0.1 mM EDTA, 0.5 mM ascorbic acid, 50 mM and 0.1 mM H_2_O_2_ in phosphate buffer (pH 7.0). The absorbance was recorded after 3 min. The APX activity was calculated using the absorbance coefficient of 2.8 mM^-1^ cm^-1^.

Glutathione peroxidase activity was determined as the decrease in absorbance at 340 nm due to the oxidation of NADPH (𝜀 = 6.22 mM^-1^ cm^-1^) according to Hopkins and Tudhope (1973). The reaction mixture consisted of 50 mM phosphate buffer (pH 8.0) containing 0.5 mM EDTA, 0.125 mM NADPH, 1 mM reduced glutathione, 0.5 U/mL glutathione reductase (Sigma-Aldrich), 300 μM tert-butyl hydroperoxide and 20 μg proteins. The reaction was monitored for 3 min.

### Glucosinolate Content

The total glucosinolates were extracted and assessed basically as described by Aghajanzadeh et al. (2014). Leaf samples (0.1 g) were boiled for 2 min in 3 ml 90% methanol. The extract was centrifuged (2500 × *g* for 2 min) and the residues were extracted again. The determination of total glucosinolate content was based on color complex formation between glucosinolates and sodium tetrachloropallade (II). The 60 μl of extract was incubated for 30 min in 1800 μl 2 mM Na_2_PdCl_4_ and absorbance of developed color was measured at 450 nm. The glucosinolate content was calculated using standard curve of sinigrin (0–3 mM; Sigma-Aldrich).

### Plant Hormone Determination

For hormone analysis *A. thaliana* and *E. salsugineum* leaf samples were collected at midday. Extraction and analysis were performed according to Dobrev and Kamínek (2002) and Dobrev and Vankova (2012). Briefly, approximately 100 mg fresh samples were homogenized and extracted with methanol/water/formic acid (15/4/1, v/v/v). The following labeled internal standards (10 pmol per sample) were added: ^2^H_6_-ABA, ^2^H_3_-PA, ^2^H_4_-SA, ^2^H_5_-JA (Olchemim, Czech Republic). Extracts were purified using SPE-C18 column (SepPak-C18, Waters, Milford, MA, USA) and separated on a reverse phase-cation exchange column (Oasis-MCX, Waters). The hormone fraction was eluted with methanol, separated by HPLC (Ultimate 3000, Dionex/Thermo Fisher Scientific, Austria) and the hormones were quantified using a hybrid triple quadrupole/linear ion trap mass spectrometer (3200 Q TRAP, Applied Biosystems/MDS SCIEX, Foster City, CA, USA). The analyses were carried out in three biological replicates. The analysis of ABA and its catabolites DPA (dihydrophaseic acid) and PA (phaseic acid); ACC (1-aminocyclopropane-1-carboxylic acid), SA (salicylic acid), jasmonic acid (JA) and jasmonate precursor *cis*-OPDA [*cis*-(+)-12-oxo-phytodienoic acid] and jasmonoyl-L-isoleucine (JA-Ile) was performed.

### Statistical Analysis

All analysis were calculated with Student’s *t*-test. Significant differences are marked at *P* ≤ 0.05.

## Results

### Production of H_2_O_2_ in Isolated Thylakoids and in Leaves

**FIGURE 1:**
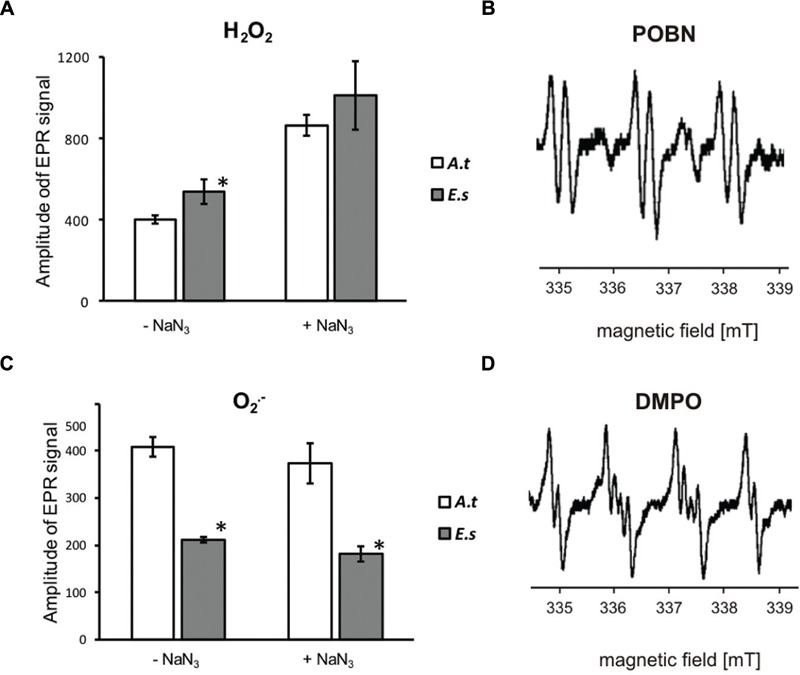
**Electron paramagnetic resonance (EPR) spin-trapping spectroscopy measurements of H_2_O_2_**(A,B)** and $O_{2}^{•–}$**(C,D)** production by illuminated thylakoids isolated from water-treated *Arabidopsis thaliana* and *Eutrema salsugineum*. (A)** The intensity of EPR signal of H_2_O_2_-derived hydroxyl radicals; **(B)** representative EPR spectra of the POBN adduct; **(C)** The intensity of superoxide anion radical measured with DMPO-OOH spin-trap; **(D)** representative EPR spectra of the DMPO adduct. The analyses were performed in the presence and absence of sodium azide (NaN_3_). ‘Asterisk’ indicates significant difference from *A*. *thaliana*. Each data point represents the mean ± SD (*n* = 3). Significant differences between the averaged H_2_O_2_ production with and without NaN_3_ in both species are not marked on the graph.

In thylakoids from *A. thaliana* and *E. salsugineum* an opposite patterns of ROS production were demonstrated by EPR method (**Figure 1**). Illuminated thylakoids of *E. salsugineum* generated more H_2_O_2_, as detected indirectly with POBN spin trap which reacts with H_2_O_2_-derived hydroxyl radical (**Figures 1A,B**), but less $O_{2}^{•–}$, as detected with DMPO spin trap (**Figures 1C,D**), in comparison to the thylakoids of *A. thaliana*. Addition of NaN_3_ strongly increased the production of H_2_O_2_, but did not significantly influence generation of $O_{2}^{•–}$ the superoxide anion radical.

**FIGURE 2:**
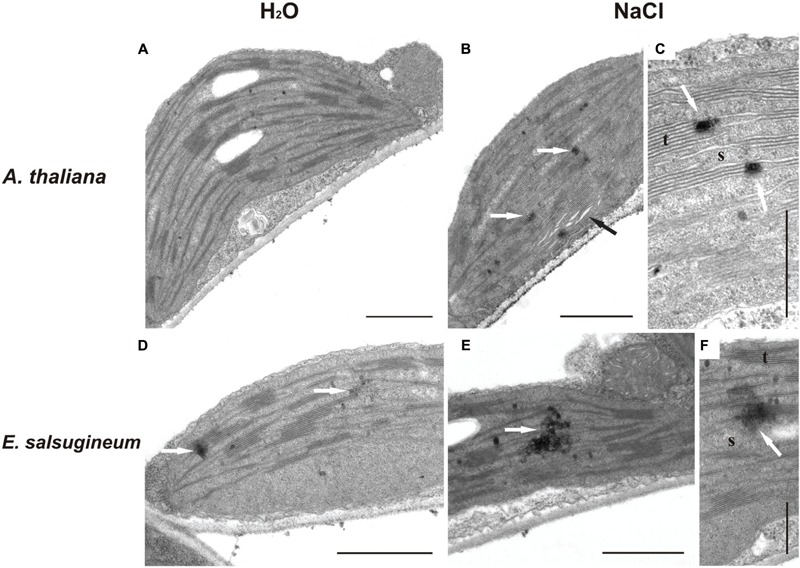
**Transmission electron microscopy (TEM) images of H_2_O_2_ accumulation in chloroplasts of *A. thaliana* and *E. salsugineum* plants treated with water and NaCl solution (0.15 and 0.3 M, respectively).** The black spots (white arrows) represent H_2_O_2_ forming electron-dense cerium perhydroxide precipitates. *A*. *thaliana* chloroplasts from water-treated **(A)** and salt-stressed plants **(B,C)**. Black arrow in **(B)** points to the swelling of thylakoids. *E*. *salsugineum* chloroplasts from water-treated **(D)** and salt-stressed plants **(E,F)**. t, thylakoids; s, stroma. Bars = 1 μm **(A,B,D,E)**; Bars = 0.5 μm **(C,F)**.

To verify unequivocally, whether a difference in H_2_O_2_ generation between the two species is significant also for chloroplasts and leaves we used several methodological approaches. The presence of H_2_O_2_ in the chloroplasts has been tested with a specific CeCl_3_ staining visualized by TEM. Chloroplasts of mesophyll cells from water-treated *A. thaliana* revealed no cerium perhydroxide deposits (**Figure 2A**), while salt stress associated H_2_O_2_ accumulation was visible as black precipitate spots (**Figure 2B**), mainly at the edges of granal thylakoids (**Figure 2C**). In contrast, in chloroplasts of *E. salsugineum* an electron-dense precipitates were detectable already in control (**Figure 2D**), while more pronounced dark spots were visible after salt stress (**Figures 2E,F**). Another difference between *A. thaliana* and *E. salsugineum* detected by TEM was that a salinity-induced thylakoid swelling was detected only in *A. thaliana* chloroplasts (**Figure 2B**), while in *E. salsugineum* chloroplasts no signs of such destruction occurred (**Figures 2E,F**). Staining controls without CeCl_3_ showed no electron-dense deposits in both species (data not shown).

**FIGURE 3:**
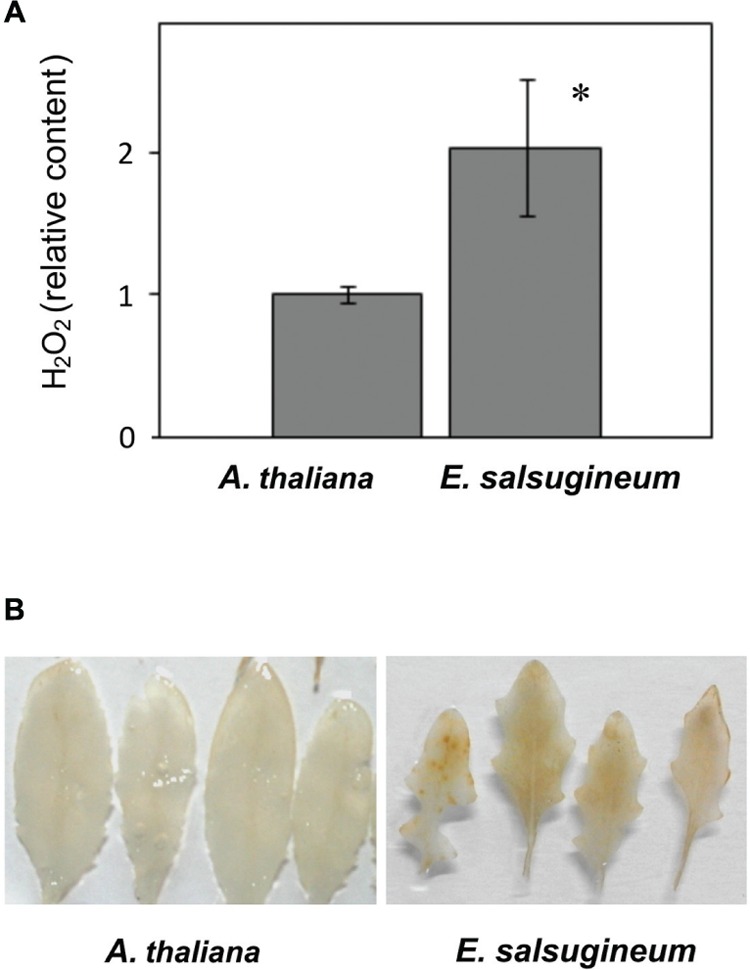
**Hydrogen peroxide (H_2_O_2_) accumulation in leaves from water-treated *A. thaliana* and *E. salsugineum* plants. (A)** Content of H_2_O_2_ in deproteinated leaf extracts determined using the Amplex Red assay. Results were normalized to the average H_2_O_2_ content in *A*. *thaliana*. Data represent the mean ± SD (*n* = 3). ‘Asterisk’ indicates significant difference. **(B)** Visualization of H_2_O_2_ accumulation assessed by DAB staining of representative leaves.

**FIGURE 4:**
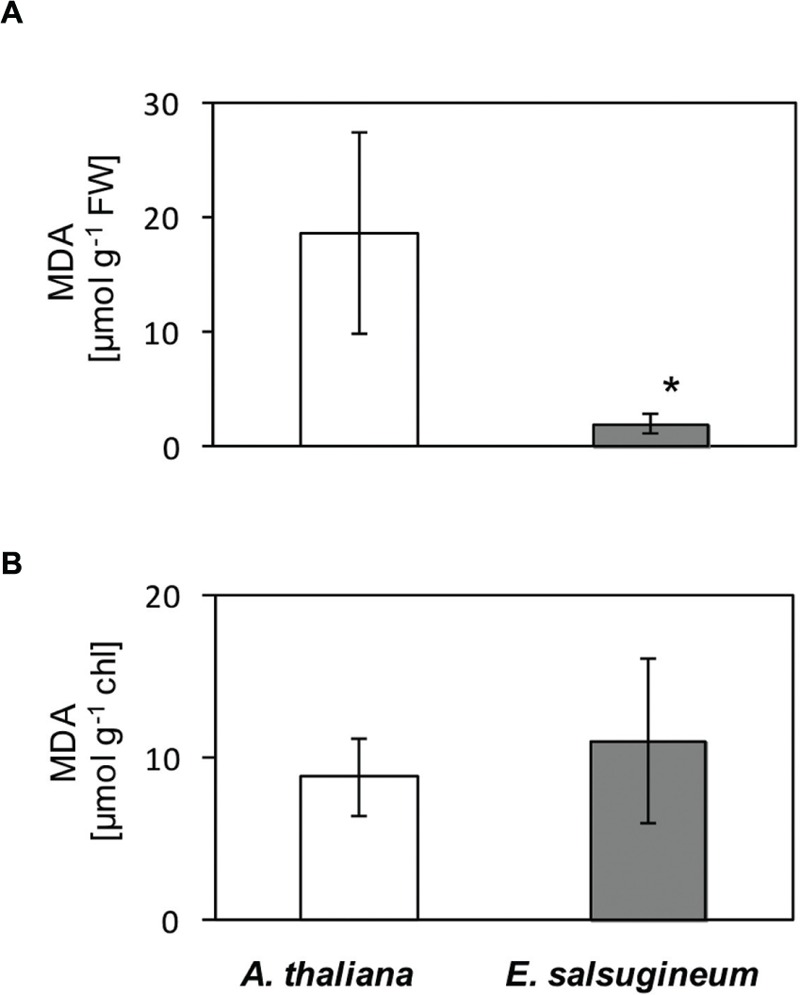
**Content of malondialdehyde (MDA) in leaves of water-treated *A*. *thaliana* and *E*. *salsugineum* plants **(A)** and isolated thylakoid membranes (TMs; **B**) assessed by high-performance liquid chromatography (HPLC).** Each data point represents the mean ± SD (*n* = 3). ‘Asterisk’ indicates significant difference from *A*. *thaliana*.

Similarly, in leaves, enhanced content of H_2_O_2_ was documented in *E. salsugineum* than in *A. thaliana* (**Figure 3A**- staining with Amplex Red, and **Figure 3B**- staining with DAB). However, in spite of the high availability of this ROS a very low level of MDA was detected in *E. salsugineum* leaves, indicating a low extent of oxidative damage to membrane lipids (**Figure 4A**). This difference between the two species disappeared when isolated TMs were compared (**Figure 4B**). On this basis we formulated a hypothesis that enhanced leakage of H_2_O_2_ from plastids keeps the antioxidant system up-regulated, thereby preadapting plants to the salinity stress.

### Comparison of Antioxidant System in *Arabidopsis* and *Eutrema*

**FIGURE 5:**
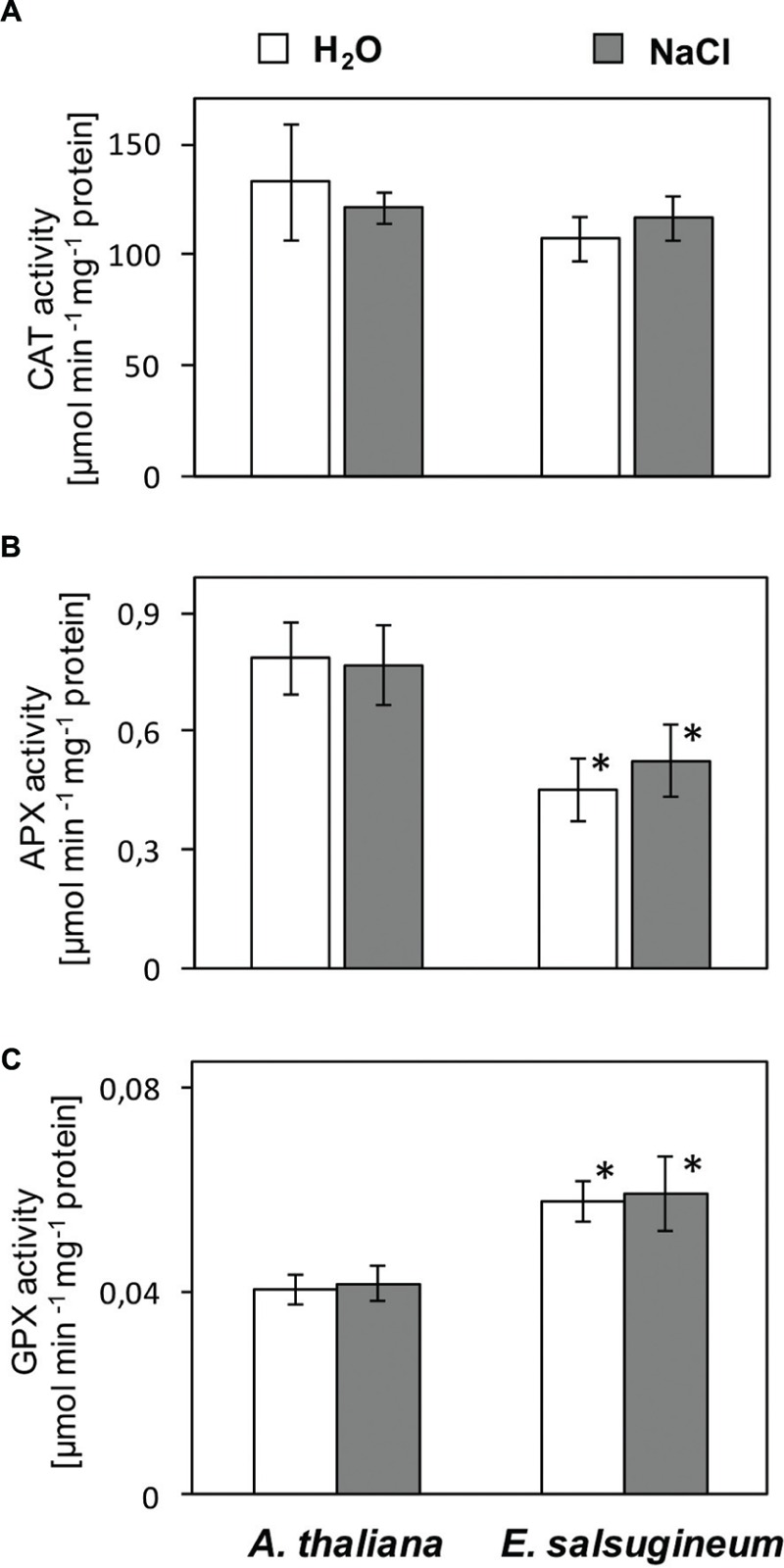
**Activities of CAT **(A)**, APX **(B)**, GPX **(C)** enzymes in the whole leaf cells of *A*. *thaliana* and *E*. *salsugineum* plants treated with water and NaCl solution (0.15 and 0.3 M, respectively).** Data represent mean ± SD (*n* = 3). ‘Asterisk’ indicates significant difference from *A*. *thaliana* from the same treatment.

The capacity of antioxidant system was compared in leaves of *A. thaliana* and *E. salsugineum* in the control conditions and after salinity stress. Due to the disturbed kinetics of enzymatic assays observed in crude leaf extracts (LEs) from *E. salsugineum*, the activities of H_2_O_2_ scavenging enzymes were analyzed in purified protein fractions. The activities of CAT were similar in both species in the control conditions and remained unchanged after salinity stress (**Figure 5A**). The activity of APX was lower in the *E. salsugineum* both in control and after salinity stress (**Figure 5B**). In contrast, the activity of thiol-dependent GPX was higher in *E. salsugineum* both in control and after a salinity stress (**Figure 5C**).

**FIGURE 6:**
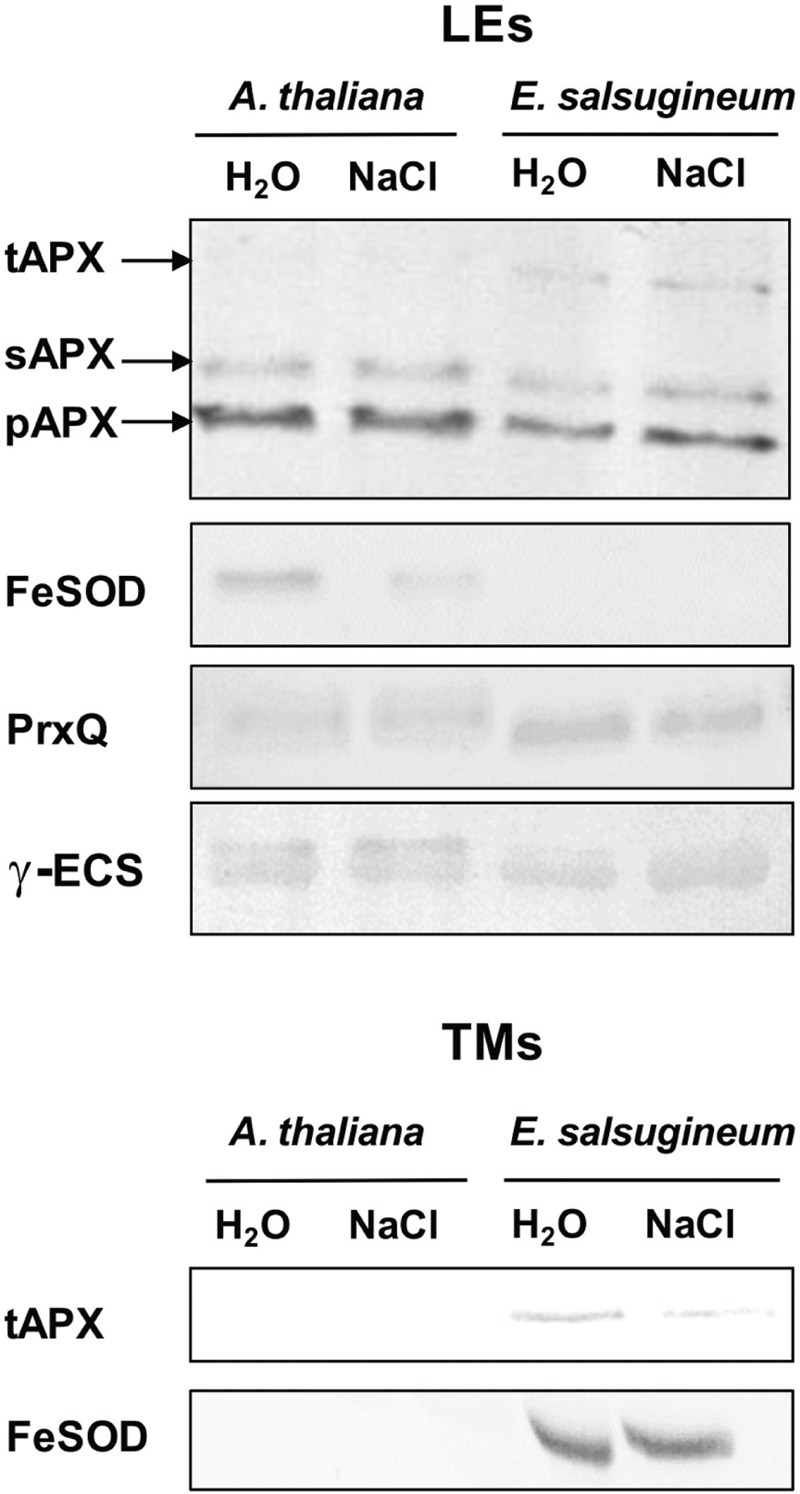
**Changes in the amount of APX (ascorbate peroxidase), FeSOD (iron superoxide dismutase), PrxQ (peroxiredoxin Q) and γ-ECS (gamma glutamylcysteine synthase) in *A*. *thaliana* and *E*. *salsugineum* plants treated with water and NaCl solution (0.15 and 0.3 M, respectively).** Immunoblotting was performed with leaf extracts (LEs) and with thylakoid membranes (TMs). For visualization of APX, FeSOD and PrxQ in LE 40, 30, and 5 μg protein per lane was loaded, respectively. For visualization of APX, FeSOD and γ-ECS in TM 10 μg chlorophyll per lane was loaded. Blots are representative for three repetitions. sAPX, stromal APX; pAPX, peroxisomal APX; tAPX, thylakoid bound APX.

Considering the chloroplastic generation of H_2_O_2_ in *E. salsugineum*, the antioxidant system in this organelle might be of particular importance for this species. Therefore, we compared the antioxidant enzymes present at TMs and in the soluble fraction (LEs; **Figure 6**). The stromal form of APX (sAPX) was present in similar amount in both species, while distribution of thylakoid bound APX (tAPX) showed a clear difference between *A. thaliana* and *E. salsugineum*. This form was much more abundant in *Eutrema* both in controls and in salinity-treated plants, while it was barely detected in *A. thaliana*. Another differences between these two species was related to FeSOD. In *E. salsugineum*, FeSOD was less abundant in LEs, while it was much more abundant in TMs, when compared with *A. thaliana* (**Figure 6**). To get an insight into the level of thiols and thiol-dependent enzymes we compared the amount of stromal peroxiredoxins Q (PrxQ) and the enzyme of reduced glutathione (GSH) synthesis, gamma glutamylcysteine synthase (γ-ECS). In *E. salsugineum* PrxQ were more abundant than in *A. thaliana*, and a slight decrease was noted after salinity treatment in both species, whilst γ-ECS was present at similar amount in both species (**Figure 6**). These pronounced differences between *E. salsugineum* and *A. thaliana* in the amounts of various ROS scavengers in chloroplasts suggest that there is a stronger need to protect photosynthetic membranes against ROS in the former species, already in the absence of stress.

### Signaling Pathways Associated with ROS

**FIGURE 7:**
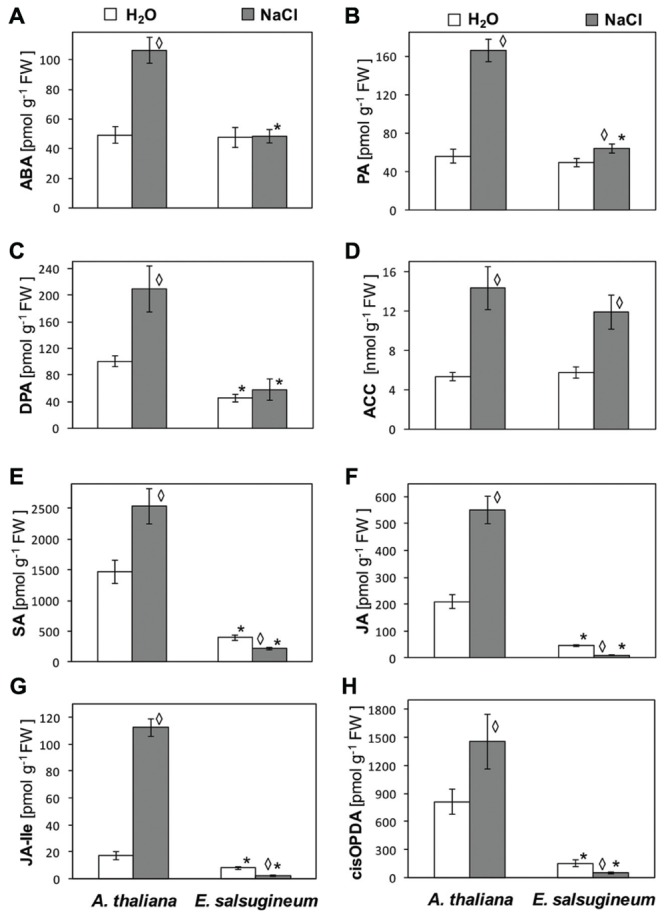
**Average content of stress-related hormones in leaves of *A*. *thaliana* and *E*. *salsugineum* plants treated with water and NaCl solution (0.15 and 0.3 M, respectively) assesed by LC–MS/MS. (A)** ABA (abscisic acid). **(B)** DPA (dihydrophaseic acid). **(C)** PA (phaseic acid). **(D)** ACC (1-aminocyclopropane-1-carboxylic acid). **(E)** SA (salicylic acid). **(F)** JA (jasmonic acid). **(G)** JA-Ile (jasmonoyl-L-isoleucine). **(H)** *cis*OPDA (*cis*-12-oxo-phytodienioc acid). Each data point represents the mean ± SD (*n* = 5). ‘Asterisk’ indicates significant difference from *A*. *thaliana* from the same treatment. ‘Diamond’ indicates a significant difference between control and salinity-treated plants.

It is well-documented that ROS signaling is interconnected with stress hormones (Baxter et al., 2014). Therefore, we analyzed the endogenous levels of stress-related hormones in *A. thaliana* and *E. salsugineum* plants grown in control conditions and changes evoked by salt-treatment. In control conditions, the concentrations of ABA and its catabolite PA were similar in leaves of both species (**Figures 7A,B**), while the level of another ABA catabolite, DPA was twofold lower in *E. salsugineum* (**Figure 7C**). Salt treatment caused an increase in ABA (twofold), DPA (twofold) and PA (threefold) in *A. thaliana*, whereas no significant changes (ABA, DPA), and a slight increase in (PA), were detected in *E. salsugineum* (**Figures 7A–C**). Concentrations of ethylene precursor ACC were similar in the two species in control conditions and underwent a similar increase due to salinity (**Figure 7D**). Concentrations of SA and jasmonates were much lower in the control *E. salsugineum* in comparison to *A. thaliana*: SA almost fourfold, JA by 4.5-fold, Ja-Ile by 2.4-fold and *cis*OPDA by 5.5-fold (**Figures 7E–H**). NaCl-treatment increased the levels of these hormones in *A. thaliana* (1.7-, 2.6-, 6.6-, 1.8-fold, respectively) but not in *E. salsugineum*, where their decreased concentrations were found (1.8-, 4-, 4-, 3.2-fold, respectively).

**FIGURE 8:**
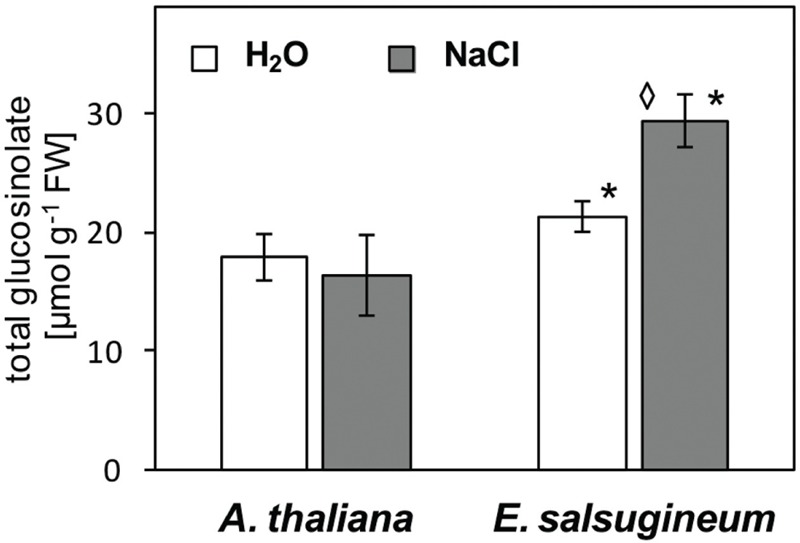
**Total glucosinolates content in leaves of *A*. *thaliana* and *E*. *salsugineum* plants treated with water and NaCl solution (0.15 and 0.3 M, respectively).** Each data point represents the mean ± SD (*n* = 3). ‘Asterisk’ indicates significant difference from *A*. *thaliana* from the same treatment. ‘Diamond’ indicates significant difference between control and salinity-treated plants.

One of the highlights of H_2_O_2_ signaling from chloroplasts seems to be enhanced synthesis of glucosinolates, as demonstrated in GO5 plants (Balazadeh et al., 2012; Sewelam et al., 2014). To check this cue in our wild type model, we measured the total concentration of leaf glucosinolates. The total level of glucosinolates was enhanced in leaves of *E. salsugineum* in comparison with *A. thaliana* in control conditions, and a stronger increase was detected in *E. salsugineum* after salinity treatment (**Figure 8**).

## Discussion

In this work, we compared a halophytic *E. salsugineum* with glycophytic *A. thaliana* in regard to the chloroplastic H_2_O_2_ signature on the salt stress tolerance. As a first step, we verified whether a chloroplastic H_2_O_2_ signal, previously reported with use of isolated TMs (Wiciarz et al., 2015), persists in the relatively undamaged organelle and tissue.

Enhanced generation of H_2_O_2_ within the chloroplasts of control and stress-treated *Eutrema* was visualized by TEM, suggesting that a higher level of H_2_O_2_ persists in the whole chloroplasts. However, it is not uniformly distributed but spotted in the proximity of granal thylakoids, were it is produced. TEM images also revealed, that NaCl treatment caused thylakoid swelling only in *A. thaliana*. Thylakoid dilation is a well-documented symptom of salinity stress in plants (Niewiadomska et al., 2011; Yamane et al., 2012; Pottosin and Shabala, 2016), while structural integrity of *E. salsugineum* chloroplasts under mild NaCl-stress was previously shown by Chang et al. (2015). This lack of swelling might be attributed to the activity of thylakoid ion channels, and/or K^+^(Na^+^)/H^+^ antiporters which control the ion homeostasis under salinity-stress (Pottosin and Shabala, 2016).

Although we did not find any direct evidence for an export of H_2_O_2_ from plastids, the total H_2_O_2_ level in leaf extracts was also elevated in *Eutrema* leaves. Hence, it is likely that a chloroplast H_2_O_2_ generation contributes considerably to this phenomenon. Our speculation is based on a well-accepted phenomenon that illuminated chloroplast is a major site of ROS production in the mesophyll cells (Asada, 2006). Moreover, a considerable leakage of H_2_O_2_ from healthy chloroplasts has been shown experimentally (Mubarakshina et al., 2010; Borisova et al., 2012).

Here, performed study revealed that an increased generation of H_2_O_2_ in halophytic species is associated by a decreased generation of $O_{2}^{•–}$. Considering a much higher reactivity of $O_{2}^{•–}$ than H_2_O_2_, this pattern of ROS production might shape a specific ROS signature important for so called stress-prepardeness of *Eutrema.* This speculation is supported by a much lower oxidative damage (MDA) in leaves of *E. salsugineum* than in *A. thaliana*. As argued by Tiwari et al. (2013), superoxide, but not H_2_O_2_, is required to evoke oxidative damage in photosynthetic membranes. This damage is mediated by the hydroxyl radical and is manifested by the appearance of carbon-centered radical. Experiments with the *flu A. thaliana* mutant and the *flu* overexpressing tAPX, enabled to demonstrate that H_2_O_2_ antagonizes the ^1^O_2_-mediated cell death response and growth inhibition, which might be particularly advantageous under stress (Laloi et al., 2007). A rapid conversion of a more harmful ROS (such as $O_{2}^{•–}$) into a longer-living and less toxic H_2_O_2_ has previously been concluded for halophytic plants on the basis of enhanced activity of superoxide dismutase (Bose et al., 2014). In halophytic *E. salsugineum* an operation of the protective electron cycling around PSII (Stepien and Johnson, 2009; Wiciarz et al., 2015) seems to support that view. Such a cycle could be beneficial for plants in several ways. Firstly, it may create a more safe electron sink in comparison to the production of superoxide and its toxic derivatives. Secondly, an abundance of H_2_O_2_ may play a signaling role.

A growing body of data indicate that H_2_O_2_ triggers defense responses in plant cells (for a recent review, see Suzuki et al., 2012; Baxter et al., 2014; Bose et al., 2014; Ismail et al., 2014). In this respect, ROS formation in chloroplast may also act indirectly via strengthening or amplifying retrograde redox signals (Galvez-Valdivieso and Mullineaux, 2010). One of the targets of ROS signaling route under stress is an up-regulation of antioxidants (Fryer et al., 2003; Geisler et al., 2006). However, the activity of a major H_2_O_2_ scavenger CAT was similar in *E. salsugineum* and *A. thaliana* in controls and in salinity-treated plants, whereas APX activity was even decreased in *E. salsugineum*. This is in agreement with numerous reports on salt cress. The transcript profiling analysis with two *E. salsugineum* ecotypes did not detect any antioxidant genes among those specifically up-regulated by salinity (Taji et al., 2004; Wong et al., 2006). Also, a proteomic data did not detect any salinity-dependent stimulation of antioxidant enzymes in this species (Pang et al., 2010). At activity level M’rah et al. (2006) previously noted that stimulation of CAT activity in *E. salsugineum* occurred only under a mild salinity treatment, not greater than 100 mM, whereas some stimulation was related to total peroxidase activity (assessed with guaiacol as electron donor).

Intriguingly, all these data suggest that a high stress resistance of *Eutrema* does not rely on the major enzymatic H_2_O_2_ scavengers of mesophyll cells. We argue, that data obtained with a partially purified (desalted) protein extracts (as used here) give a more precise information about changes in the enzymatic activities in comparison to the data typically obtained with crude extracts. The latter are strongly influenced by a different availability of low molecular compounds, such as ascorbate in case of APX, and glutathione in case of GPX. Indeed, an increased pool of reduced ascorbate and of total glutathione was found due to salinity in *Eutrema*, but not in *Arabidopsis* (Wiciarz et al., 2016). This feature might be important in the context of H_2_O_2_ signaling. As suggested by Bose et al. (2014), decreased activities of a major H_2_O_2_ scavengers allow for a persistence of ROS signal in the cells. A fine-tuning of specific antioxidants seems to be more important for stress tolerance than a bulk changes in the antioxidant status (Jiang et al., 2007; Pang et al., 2010). We are, however, aware that a relation between ROS production and scavenging is highly dynamic while a situation reported here for *Eutrema* relates only to certain time point. But, on the other hand, it was verified with use of several methods and plants sets to be considered as physiologically relevant.

Among the enzymatic H_2_O_2_ scavengers only GPX was enhanced in *E. salsugineum*. This points to the importance of redox active thiols. GPXs reduce not only H_2_O_2_ but also organic hydroperoxides using reducing power of GSH. Hence, they may contribute to the dramatically reduced MDA level. A noteworthy stimulation of antioxidant defense associated with glutathione has previously been revealed in *E. salsugineum* by transcript profiling of Gong et al. (2005). This was represented by GPXs (*GPX2* and *GPX6*), glutaredoxins and glutathione-*S*-transferases (*GSTZ1* and *GSTU7*). Four out of eight GPXs found in *E. salsugineum* have been shown to respond to salt and osmotic stresses at both transcript and protein level (Gao et al., 2014). It is worthy to note here, that GPX may serve as a specific sensor and transducer of H_2_O_2_ signal. A dual function of GPX associated with both scavenging of ROS and transferring the H_2_O_2_ signal has been pointed out repeatedly (Delaunay et al., 2002; Miao et al., 2006). Moreover, a specific activation of *GPX1* promoter in tobacco by a chloroplast-generated H_2_O_2_ was documented by Avsian-Kretchmer et al. (2004). Whereas, study with *Arabidopsis gpx3* mutant demonstrated that GPX3 participates in the ABA and H_2_O_2_ signaling pathway regulating the stomatal aperture and resistance to drought (Miao et al., 2006).

Considering an enhanced generation of H_2_O_2_ and changed $O_{2}^{•–}$/H_2_O_2_ ratio in chloroplasts, a major role might be attributed to the anti-oxidative protection within this organelle. Indeed, TMs of *Eutrema* appeared to be better protected against ROS in comparison with *Arabidopsis*, as indicated by a higher abundance of tAPX and FeSOD. This corresponds with enhanced level of *FeSOD* transcripts in the control *E. salsugineum*, reported by Taji et al. (2004). In regard to chloroplast stroma, we found an enhanced amount of PrxQ in *E. salsugineum*. These above-mentioned features of chloroplast antioxidant system are constitutive in *E. salsugineum*, which supports earlier conclusions on the stress preparedness of this species (Inan et al., 2004; Taji et al., 2004; Gong et al., 2005; Wong et al., 2006). They are also in line with the view that components of redox homeostasis in chloroplasts are crucial for salinity tolerance (Niewiadomska and Wiciarz, 2015; Uzilday et al., 2015). So far, recognized chloroplast redox players highly engaged in *E. salsugineum* are: adenosine 5′-phosphosulfate reductase involved in sulfate assimilation (Gong et al., 2005); thioredoxin CDSP32 (M’rah et al., 2007); 2-cys peroxiredoxin BAS1 (Gao et al., 2009) and plastid terminal oxidase (PTOX), an enzyme of chlororespiratory pathway (Stepien and Johnson, 2009; Wiciarz et al., 2015). In addition to these, also proteins associated with the Calvin-Benson cycle, such as a more active Rubisco (Wiciarz et al., 2015) and a more abundant glyceraldehyde 3-phosphate dehydrogenase (Pang et al., 2010; Chang et al., 2015) are likely to contribute to the improved redox balance in *E. salsugineum* by efficient recycling of ADP and NADP^+^.

A very weak response to salinity stress at the level of tested antioxidants may raise a question: whether *E. salsugineum* (and *A. thaliana*) senses the salinity stress at all? This has been clarified by analysis of stress hormones. A massive increase in stress-related hormones (ABA, SA, JA, and ethylene precursor) was detected in salinity-treated *A. thaliana*. These hormones are responsible for several stress effects in plants, among them stomatal closure and retardation of growth, although, their action is quite complex and may lead either to survival or to cell death. In contrast, in *Eutrema* the only indicator of salinity-evoked stress was a stimulation of ethylene synthesis. Although, some enhancement in ABA catabolite PA, may suggest a previous ABA induction. Earlier study of Ellouzi et al. (2014) documented that levels of ABA, JA, and ACC were increased in *E. salsugineum* after 2 days of salt stress, while 400 mM NaCl treatment for more than 5 days caused minimal changes in ABA and JA concentrations (Taji et al., 2004; Arbona et al., 2010). In *E. salsugineum* the levels of lipid-based active jasmonates (JA and JA-Ile) were low and even decreased due to salinity-treatment. This effect seems to be closely related to salt-resistance of *Eutrema*. As shown by Ismail et al. (2014), an interplay between ABA and JA is of particular importance for plant’s survival or death under salinity stress. A comparison of the two *Vitis* species differing in salt-stress tolerance revealed that in the more salt-tolerant *Vitis rupestris* ABA accumulated early and strongly suppressed the formation of JA-Ile, while in the salt-sensitive *V. vinifera* the accumulation of ABA was delayed and correlated which allow for accumulation of JA-Ile up to the high levels. A suppression of jasmonates in *E. salsugineum* might also be explained in accordance with the data obtained with AtGO5 mutant overproducing H_2_O_2_ in chloroplasts, in which a stimulated expression of several repressors of JA signaling pathway has occurred (Balazadeh et al., 2012). A very weak response to salinity at the level of ABA in *E. salsugineum* in contrast to *A. thaliana* is surprising in view of data on a higher expression of genes for ABA-biosynthesis and signaling (Taji et al., 2004; Gong et al., 2005; Wu et al., 2012) and an increased number of genes involved in ABA synthesis pathway in *Eutrema* genome (Wu et al., 2012). Possibly, in *E. salsugineum* the increased level of ABA is confined to the early stage of development or this hormone is very rapidly modulated in response to stress (indicated by slight increase in PA). A weak response of stress hormones to salinity in *E. salsugineum* supports the earlier conclusion of Hou and Bartels (2015), that comparing with *A. thaliana* the higher NaCl levels (600 mM) are required in *E. salsugineum* to trigger some defense reactions, such as increased transcription of two aldehyde dehydrogenase genes.

On the other hand, the importance of glucosinolates for stress-resistance of *E. salsugineum* is depicted by their enhanced level and a strong salinity-dependent increase. A great variation in the patterns of aliphatic, aromatic, and indole glucosinolate was documented in *E. salsugineum*, depending on the organ and developmental stage (Pang et al., 2012). However, a precise role of these compounds in stress tolerance is not clarified yet. An up-regulation of the synthesis of indole glucosinolates and phytoalexin camalexin was found as a specific feature of increased H_2_O_2_ signaling from chloroplasts in studies with *At*GO5 mutant made by Balazadeh et al. (2012) and Sewelam et al. (2014). However, *E. salsugineum* does not produce camalexin, but wasalexins A and B and methoxybrassenin B (Pedras and Adio, 2008). Intriguingly, as another support for a constitutive stress-preparedness of *Eutrema*, the three phytoalexins (indolyl-3-acetonitrile, caulilexin C, and arvelexin) are found as phytoanticipins, i.e., they are constitutively produced in this species (Pedras and Adio, 2008).

## Conclusion

In this paper we provided evidence for a specific pattern of ROS formation and accumulation in halophytic *Eutrema* in comparison to glycophytic *Arabidopsis*. In concert with that, a pronounced changes in the antioxidant system were revealed in *E. salsugineum*, such as a decreased activity of APX, an increased activity of GPX, an increased level of PrxQ, and the presence of thylakoid-bound forms of FeSOD and APX. All these cues allow for enhanced H_2_O_2_ signaling from chloroplasts of *E. salsugineum* already in the control conditions, at decreased level of oxidative damage in the same time. This signaling led to enhanced level of glucosinolates in *Eutrema* and decreased levels of stress hormones (SA and jasmonates). Salinity-stress evoked a strong up-regulation of all tested stress hormones in *A. thaliana* whereas in *E. salsugineum* only a stimulation in ethylene synthesis and ABA catabolism was noted. On this basis we hypothesize that H_2_O_2_ signaling is engaged in the halophytic species to modulate a hormonal responses in such a way to minimize the triggering of jasmonate pathway which leads to cell death.

## Author Contributions

MP performed analysis of antioxidants, staining and quantitation of H_2_O_2_, determination of glucosinolates, participated in TEM analysis and in preparation of the manuscript. MW measured MDA, participates in the analysis of antioxidants and ROS. IJ measured ROS with EPR. MK-K performed TEM analysis. PD and RV measured stress hormones, and RV participated in writing of the text. EN designed the experiment and wrote the manuscript. All authors have read and approved the manuscript.

## Conflict of Interest Statement

The authors declare that the research was conducted in the absence of any commercial or financial relationships that could be construed as a potential conflict of interest.
